# TLR8 in the Trigeminal Ganglion Contributes to the Maintenance of Trigeminal Neuropathic Pain in Mice

**DOI:** 10.1007/s12264-020-00621-4

**Published:** 2020-12-23

**Authors:** Lin-Xia Zhao, Ming Jiang, Xue-Qiang Bai, De-Li Cao, Xiao-Bo Wu, Jing Zhang, Jian-Shuang Guo, Tong-Tong Chen, Juan Wang, Hao Wu, Yong-Jing Gao, Zhi-Jun Zhang

**Affiliations:** 1 Department of Human Anatomy, School of Medicine, Nantong University, Nantong, 226001 China; 2 Institute of Pain Medicine, Institute of Nautical Medicine, Nantong University, Nantong, 226019 China; 3 Department of Otolaryngology Head Neck Surgery, The Affiliated Hospital of Nantong University, Nantong, 226001 China; 4 Co-innovation Center of Neuroregeneration, Nantong University, Nantong, 226001 China

**Keywords:** TLR8, ERK, p38, Pro-inflammatory cytokine, Trigeminal ganglion, Trigeminal neuropathic pain, Mouse

## Abstract

Trigeminal neuropathic pain (TNP) is a significant health problem but the involved mechanism has not been completely elucidated. Toll-like receptors (TLRs) have recently been demonstrated to be expressed in the dorsal root ganglion and involved in chronic pain. Here, we show that TLR8 was persistently increased in the trigeminal ganglion (TG) neurons in model of TNP induced by partial infraorbital nerve ligation (pIONL). In addition, deletion or knockdown of *Tlr8* in the TG attenuated pIONL-induced mechanical allodynia, reduced the activation of ERK and p38-MAPK, and decreased the expression of pro-inflammatory cytokines in the TG. Furthermore, intra-TG injection of the TLR8 agonist VTX-2337 induced pain hypersensitivity. VTX-2337 also increased the intracellular Ca^2+^ concentration, induced the activation of ERK and p38, and increased the expression of pro-inflammatory cytokines in the TG. These data indicate that TLR8 contributes to the maintenance of TNP through increasing MAPK-mediated neuroinflammation. Targeting TLR8 signaling may be effective for the treatment of TNP.

## Introduction

The sensation of nociceptive stimuli is mediated by primary sensory neurons in the dorsal root ganglion (DRG) and trigeminal ganglion (TG), which transmit noxious information to the brain *via* the spinal cord and medulla oblongata, respectively. Painful sensation from the orofacial area is relayed in the TG, which has three major branches: the ophthalmic (V1), maxillary (V2), and mandibular nerves (V3). Trigeminal neuropathic pain (TNP) is usually a result of injury or disease of one or more nerve branches (usually V2 and/or V3) and can be triggered by subtle sensory stimuli to the affected side of the face, such as light mechanical touch, brushing teeth, and chewing [1]. TNP is a chronic state and difficult to treat in clinical practice. Understanding the pathophysiology of TNP is essential for pain management.

In recent years, increasing evidence has revealed that neuroinflammation is involved in the pathological process of neuropathic pain including TNP [2–5]. After peripheral nerve injury, neuroinflammation occurs at different anatomical locations on the pain transmission pathway, including the TG/DRG and medulla oblongata/spinal cord, which facilitates peripheral and central sensitization [6–9]. Among inflammatory mediators, cytokines such as tumor necrosis factor (TNF)-α, interleukin (IL)-1β, and IL-6 are well demonstrated to be increased in the peripheral nervous system after nerve injury and enhance neuronal excitability [7, 10–12]. Inhibiting these cytokines using neutralizing antibodies or RNA interference inhibits the pain behavior in several neuropathic pain models [7].

Toll-like receptors (TLRs) play a vital role in the innate and adaptive immune responses [13]. After binding with ligands, TLRs initiate and regulate the inflammatory response *via* the release of cytokines [14, 15]. The family of TLRs has 12 functional members in mice (TLR1-TLR9 and TLR11-TLR13). Unlike other TLRs, TLR3, 7, 8, and 9 are localized in intracellular compartments of the immune system [14, 16]. However, the evidence shows that TLR7 is localized on the membrane of DRG neurons and regulates itch transmission [17]. In addition, a recent report confirmed that TLR7 is expressed in injured DRG neurons and contributes to neuropathic pain *via* activating the NF-κB signaling pathway [18]. Recently, TLR9 has been found in macrophages in the DRG and contributes to paclitaxel-induced neuropathic pain in mice [19]. The *Tlr8* and *Tlr7* genes show high homology to each other and are both located on the X chromosome. However, TLR8 is located in the intracellular endoplasmic reticulum (ER), endosomes, and lysosomes of DRG neurons, and plays an important role in the pathogenesis of spinal nerve injury-induced neuropathic pain [20]. How TLR8 is expressed in the TG after infraorbital nerve injury and whether TLR8 is involved in the TNP have not been investigated.

The mitogen-activated protein kinases (MAPKs), which include extracellular signal-regulated kinase (ERK), p38, and c-Jun N-terminal kinase (JNK), have been reported to contribute to neuroinflammation and chronic pain [21]. In the DRG, the activation of MAPKs including ERK and p38 increases the expression of IL-1β and IL-6, and is involved in post-operative and inflammatory pain [22, 23]. Our earlier study showed that a TLR8 agonist activates ERK in DRG neurons and induces pain hypersensitivity [20]. In this study, we used the partial ligation of the infraorbital nerve (pIONL) model to investigate the role of TLR8 in the TG during the pathogenesis of TNP. We found that TLR8 neurons facilitate TPN *via* increasing intracellular Ca^2+^, activating ERK and p38, and further increasing the expression of pro-inflammatory cytokines in the TG after infraorbital nerve injury.

## Materials and Methods

### Animals and Surgery

ICR male mice weighing 26 g–30 g were provided by the Experimental Animal Center at Nantong University. *Tlr8*^−/−^ mice were developed by Cyagen Co. (Suzhou, China). *Tlr7*^−/−^ mice were purchased from the Jackson Laboratory (stock number 008380). All mice were housed in standard clear plastic cages under controlled ambient temperature (22 °C–24 °C) with a reversed 12:12 h dark/light cycle, and allowed *ad libitum* access to water and food. The experimental and surgical procedures were reviewed and approved by the Animal Care and Use Committee of Nantong University. Animal treatments were performed in accordance with the guidelines of the International Association for the Study of Pain.

The pIONL surgery was as described by Zhang *et al*. [24, 25]. In brief, mice were anesthetized with sodium pentobarbital (40 mg/kg–50 mg/kg, i.p.), and then the oral cavity was opened to locate the tendon of the left masseter muscle on the upper wall of oral cavity in the supine position. In front of the tendon, an incision (1 mm) was made to expose the infraorbital nerve. The TNP model was established by ligating one-half of the infraorbital nerve (ION) with 6-0 silk suture. The mucous membrane of the incision was closed with Tissue Adhesive (Vetbond; 3M, USA). The sham operation comprised an incision on the mucous membrane without damaging the ION.

### DNA Extraction and Genotyping

A piece ~2 mm in diameter was cut from the ear each *Tl8*^−/−^ or *Tlr7*^−*/*−^ mouse under brief anesthesia with isoflurane, and then used to extract DNA with the phenol-chloroform method. The primers for genotyping *Tlr8*^−/−^ mice were as follows: forward: 5′-GCA GTT GAC GAT GGT TGC ATT-3′, and reverse: 5′-TGA CGT GCT TTT GTC TGC TG-3′. A 50 μL reaction volume of PCR amplification buffer was used, including 200 ng DNA, 25 μL 2×Taq PCR MasterMix (Tiangen Biotech), and 1.0 μmol/L TLR8 genotyping primers. Reactions were initially denatured at 95 °C for 5 min, 35 cycles at 95 °C for 30 s, 60 °C for 30 s, 72 °C for 30 s, and then final extension at 72 °C for 2 min. The genotyping protocol for *Tlr7*^−/−^ mice was as described by the Jackson Laboratory. The primers included the common forward primer for olMR8628: 5′-AGG GTA TGC CGC CAA ATC TAA AG-3′, the wild-type reverse primer for olMR8629: 5′-ACC TTT GTG TGC TCC TGG AC-3′, and the mutant reverse primer for olMR8630: 5′-TCA TTC TCA GTA TTG TTT TGC C-3′. For PCR amplification, 200 ng DNA was used in a 50 μL reaction volume containing 25 μL 2×Taq PCR MasterMix (Tiangen Biotech), and 0.5 μmol/L primers for olMR8628, olMR8629, and olMR8630. Reactions were initially denatured at 94 °C for 2 min, 10 cycles at 94 °C for 20 s, 65 °C for 15 s (0.5 °C decrease per cycle), 68 °C for 10 s, and then 28 cycles at 94 °C for 15 s, 60 °C for 15 s, 72 °C for 10 s, and then final extension at 72 °C for 2 min. Amplicons were separated on 1.5% agarose gel, stained with DuRed (Biotium), and photographed with the GelDoc-It Ts Imaging System (UVP, USA).

### Drugs and Administration

VTX-2337 was from Active Biochem (Hong Kong, China). *Tlr8* siRNA and negative control siRNA (NC siRNA) were designed by RiboBio [20]. RVG-9R peptide was from AnaSpec (USA). For peri-infraorbital nerve injection, the *Tlr8* siRNA (2 µg) or NC siRNA was mixed with RVG-9R peptide (molar ratio 1:10) [20, 26]. Intra-TG injection was performed with a 29 G syringe (Becton, Dickinson and Co., USA). After deep anesthesia with isoflurane, the head of the mouse was held in one hand. The tip of the needle was then inserted through the infraorbital foramen, infraorbital canal, and foramen rotundum, and finally was positioned in the TG [27]. Different doses (10, 50, and 100 ng) of VTX-2337 (5 μL) were slowly injected.

### Facial Pain Behavioral Test

The behavioral test environment was kept at 22 °C–24 °C and 40%–60% humidity. Before facial behavioral tests, the mice were habituated to the behavioral test cage for 30 min every day for 3 days in the test environment. Von Frey filaments (0.02 g and 0.16 g) were used to stimulate the whisker pad innervated by the ION, and the responses of the mice were recorded. The filaments were applied 3 times on the ipsilateral whisker pad. The mean nocifensive behavior score of 3 measurements was calculated according to the following criteria: score 0, no response; score 1, exploratory behavior - the mouse detected the von Frey filament; score 2, slight withdrawal response - the mouse slowly moved its face back from the stimulation; score 3, quick and intense withdrawal response with lifting of the paw; score 4, wiping the face with the forepaw < 3 times in the stimulated facial area; score 5, wiping the face with the forepaw > 3 times in the stimulated facial area [28, 29].

### RNA Collection and Real-Time RT-PCR

Using the manufacture’s protocol of TRIzol reagent (Invitrogen, Carlsbad, CA, USA), the total RNA in the TG was collected. The quality and quantity of RNA was checked on a NanoDrop spectrophotometer (Thermo Fisher Scientific, USA). The cDNA was reverse-transcribed from RNA using a First Strand cDNA Synthesis Kit (Takara, Japan). Real-time PCR was performed in A&B Applied Biosystems apparatus using SYBR Green Master Mix (Vazyme, China). The following primers were used in the PCR reaction: TLR8 forward: 5′-ACC TGA GCC ACA ATG GCA TTT AC-3′, TLR8 reverse: 5′-TTG CCA TCA TTT GCA TTC CAC-3′; TNF-α forward: 5′-GTT CTA TGG CCC AGA CCC TCA C-3′, TNF-α reverse: 5′-GGC ACC ACT AGT TGG TTG TCT TTG-3′; IL-1β forward: 5′-TCC AGG ATG AGG ACA TGA GCA C -3′, IL-1β reverse: 5′-GAA CGT CAC CCA GCA GGT TA-3′; IL-6 forward: 5′-CCA CTT CAC AAG TCG GAG GCT TA-3′, IL-6 reverse: 5′-CCA GTT TGG TAG CAT CCA TCA TTT C-3′; GAPDH forward: 5′-AAA TGG TGA AGG TCG GTG TGA AC-3′, GAPDH reverse: 5′-CAA CAA TCT CCA CTT TGC CAC TG-3′. The conditions of PCR amplification were set at 95 °C for 30 s, and then 40 cycles (5 s for 95 °C and 30 s for 60 °C). The PCR data were analyzed with StopOne Software v2.3. Melting curves were used to assess the specificity of PCR products. Quantification was performed by normalizing the cycle threshold (Ct) values with GAPDH Ct and analyzed with the 2^−ΔΔCt^ method.

### Immunofluorescence

After deep anesthesia with the isoflurane, the mice were transcardially perfused with 0.01 mol/L PBS followed by 4% paraformaldehyde in 0.01 mol/L PBS. The TG was dissected carefully, postfixed in fixative overnight, and then left in 30% sucrose in 0.01 mol/L PBS for 2 days. The TG was embedded in OCT solution and then cut into 15-μm sections on a cryostat (Leica, Germany). The sections were processed for immunofluorescence as we described previously [20]. In brief, the sections were blocked by 5% donkey serum in PBS at room temperature for 1 h. Subsequently, the sections were incubated with primary antibodies against TLR8 (rabbit, 1:500, BosterBio), Tuj1 (mouse, 1:2000, R&D Systems), CD68 (rat, 1:1000, AbD Serotec), ATF3 (mouse, 1:500, Santa Cruz), CGRP (mouse, 1:1000, Sigma-Aldrich), and NF200 (mouse, 1:500, Cell Signaling Technology) in a humidified box at 4 °C overnight. Following three rinses with 0.01 mol/L PBS, the sections were further incubated with the secondary antibodies donkey anti-rabbit Cy3 (1:1000, Jackson ImmunoResearch), donkey anti-rat Alexa Fluor 488 (1:1000, Jackson ImmunoResearch), donkey anti-mouse Alexa Fluor 488 (1:1000, Jackson ImmunoResearch), and IB4-FITC (1:200, Sigma-Aldrich). The fluorescence signals were checked and captured under a fluorescence microscope (Nikon Eclipse Ni-E, Japan). The fluorescence images were analyzed with ImageJ (NIH, USA).

### Western Blot

The TGs from WT and *Tlr8*^−/−^ mice were homogenized in lysis buffer containing protease and phosphatase inhibitors (Sigma-Aldrich). The protein concentration was checked using the BCA Protein Assay (Pierce Biotechnology, USA). Thirty micrograms of protein was loaded in each lane of SDS-PAGE gel, and then transferred to polyvinylidene fluoride membrane. After blocking by 5% skim milk, the membrane was incubated with the primary antibody against pERK (rabbit, 1:500, Cell Signaling Technology), ERK (rabbit, 1:500, Cell Signaling Technology), pp38 (rabbit, 1:500, Cell Signaling Technology), or p38 (rabbit, 1:500, Cell Signaling Technology). The membrane was further incubated with IRDye 800CW secondary antibody (goat-anti-rabbit, 1:10000, LI-COR) and the images captured with an Odyssey CLx system (Odyssey, USA). The sizes of bands were evaluated by the pre-stained protein marker (Thermo Fisher Scientific), and the intensity of bands was calculated by ImageJ.

### HEK293 Cell Culture and Transfection

HEK293 cells were cultured in high-glucose Dulbecco’s modified Eagle’s medium (Bio Whittaker Europe, Vervier, Belgium) with 10% (vol/vol) fetal calf serum (PAA, Linz, Austria) and 0.5% penicillin/streptomycin at 37 °C in a humidity-controlled incubator with 5% (vol/vol) CO_2_. Cells were transfected with the TLR8 or control plasmids using Lipofectamine 2000 (Invitrogen, Netherlands) at 80% confluence. Transfected cells were cultured in the same growth medium for 36 h before Ca^2+^ measurements.

### TG Neuron Culture

After anesthesia using isoflurane, the cranium and brain of mice (4–6 weeks old) were quickly removed, and the TGs rapidly collected [30]. The meninges and connective tissue on the TG were carefully stripped in an ice-cold oxygenated balanced salt solution (BSS, in mmol/L: 125 NaCl, 3 KCl, 26 NaHCO_3_, 1.25 NaH_2_PO_4_, 10 glucose, 2.4 CaCl_2_, 1.2 MgCl_2_, 5 HEPES, pH 7.2 and osmolarity 300 mOsm), and each TG was shredded with scissors. The TG tissue was kept at 37 °C for 90 min in oxygenated aCSF which contained collagenase (3.0 mg/mL, Roche) and dispase-II (2.4 units/mL, Roche), and then washed twice with standard aCSF. TG neurons were separated mechanically with polished glass pipettes, and cultivated on adhesive coverslips (diameter, 13 mm) in 24-well plates. The dissociated TG neurons in each well were incubated in aCSF at 37 °C (humidified 95% O_2_ and 5% CO_2_). After 24 h incubation, TG neurons were used for Ca^2+^ imaging measurements.

### Ca^2+^ Imaging

HEK 293 cells or TG neurons were loaded with Fura-2AM (2 µmol/L, Molecular Probes) mixed with 0.02% pluronic (Life Technologies) for 90 min at room temperature in darkness. Coverslips with HEK 293 cells or TG neurons were washed with BSS and then placed on an inverted fluorescence microscope (Olympus IX73, Japan). The fluorescence emission at 510 nm with excitation at 340 nm and 380 nm was detected at 2-s intervals using a computer-controlled F4500 fluorescence spectrophotometer (Hitachi, Japan). Wavelength selection and the timing of excitation and acquisition of images were controlled using the Metafluor program (Molecular Devices). Digital images were stored on hard disk for off-line analysis. The ratio of fluorescence intensities (λ340/λ380) at these two wavelengths was recorded as the relative level of intracellular Ca^2+^.

### Quantification and Statistics

All results are shown as mean ± SEM. The behavioral data were analyzed by two-way repeated measures (RM) ANOVA followed by the Bonferroni test as the *post-hoc* multiple comparison analysis. For the analysis of TLR8^+^ neurons in the TG, immunofluorescent images of TG were captured, and the number of TG neurons was counted using a computer-assisted imaging analysis system (ImageJ). For western blot, the density of specific bands was measured with ImageJ, and the intensity of the background was subtracted in each lane. The levels of pERK and pp38 were normalized to total ERK and p38, respectively. Differences between groups were compared using one-way ANOVA followed by the Bonferroni test or using Student’s *t*-test if only 2 groups were compared. The criterion for statistical significance was set at *P* < 0.05.

## Results

### TLR8 Expression is Increased in TG Neurons After pIONL-Induced TNP

**Fig. 1:**
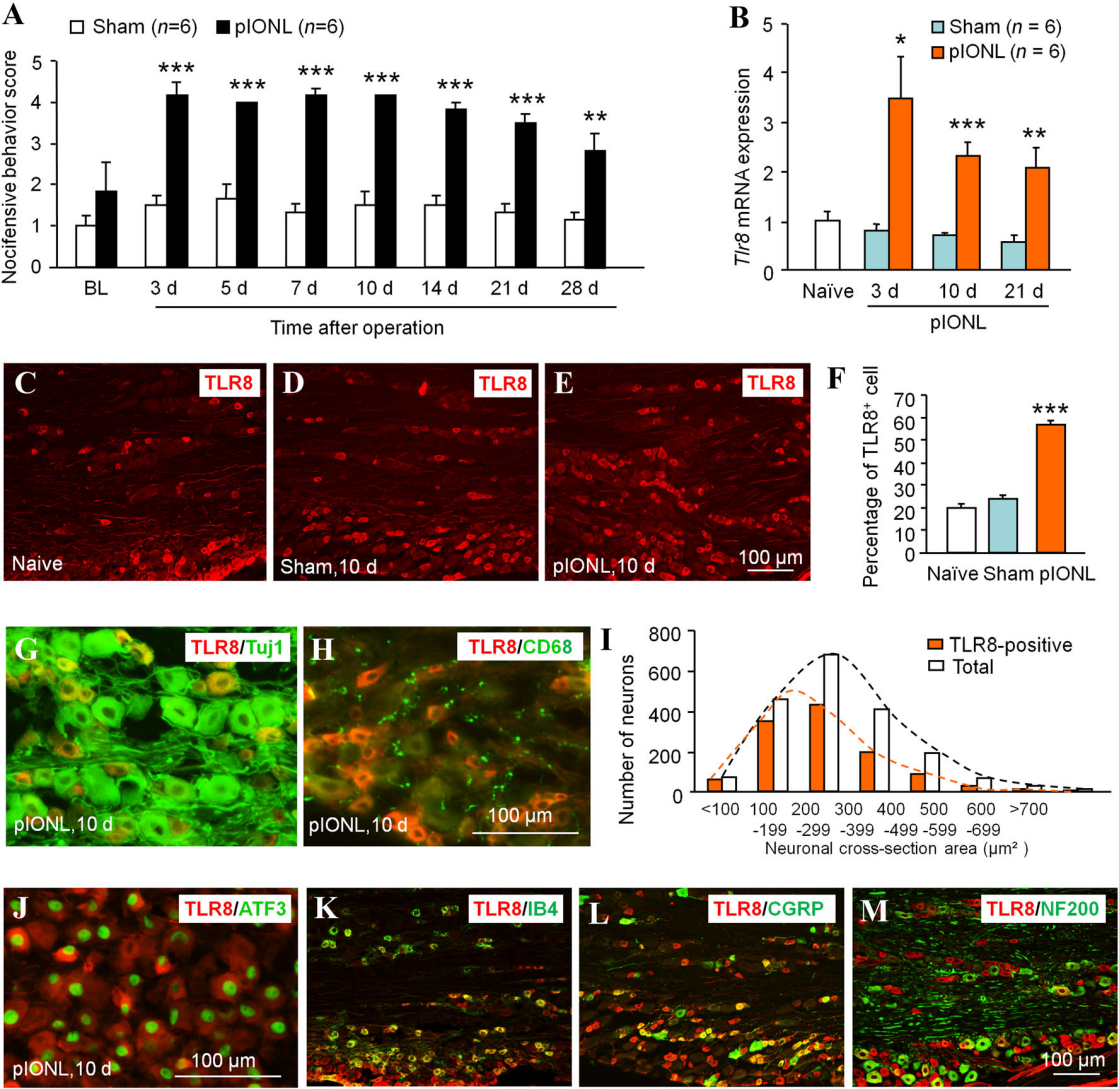
pIONL causes mechanical allodynia of the ipsilateral facial skin, and increases TLR8 expression in the TG. **A** Time-course of facial nocifensive behavior score stimulated by a 0.02 g von Frey filament in sham and pIONL mice (**P < 0.01, ***P < 0.001 *vs* corresponding sham group, two-way RM ANOVA followed by *post-hoc* Bonferroni test; *n = *6 mice/group). **B** Real-time PCR showing the expression of *Tlr8* mRNA in the TG of naïve, sham, and pIONL mice (*P < 0.05, **P < 0.01, ***P < 0.001 *vs* sham, Student’s *t*-test; *n = *6 mice/group). **C–E** Representative TLR8 immunofluorescent images showing the distribution of TLR8^+^ neurons in the TG of naïve (**C**), sham (**D**), and pIONL 10 days (**E**) mice. **F** Percentages of TLR8^+^ cells in the TG of naïve, sham, and pIONL mice (***P < 0.001 *vs* sham, one-way ANOVA followed by the Bonferroni test; *n = *3 mice/group). **G**, **H** Double immunostaining of TLR8 with Tuj1 (**G**) and CD68 (**H**). **I** Cell-size distribution of TLR8^+^ neurons and total neurons in the TG 10 days after pIONL. **J–M** Double-stained immunofluorescent images showing the co-localization of TLR8 with ATF3 (**J**), IB4 (**K**), CGRP (**L**), and NF200 (**M**) in the TG.

Before and after pIONL, we used the numerical value of the facial nocifensive behavior score, which was induced by a 0.02 g (or 0.16 g) von Frey filament stimulation of the whisker pad, to evaluate the degree of TNP [28]. Behavioral data showed that pIONL increased the nocifensive behavior score stimulated with a 0.02 g von Frey filament (Fig. 1A), starting from day 3 and lasting for > 28 days after the operation (Fig. 1A). The scores for a 0.16 g filament were similar (data not shown). We then checked the expression of TLR8 in the TG after pIONL. Real-time PCR showed that the expression of *Tlr8* mRNA was increased at days 3, 10, and 21 after pIONL (Fig.1B). Immunostaining showed a few TLR8^+^ neurons scattered in the TG of naïve and sham-operated mice, but this was dramatically increased in pIONL mice at day 10 (Fig. 1C–E). Statistical data showed that TLR8^+^ cells were significantly increased at pIONL day 10 (Fig. 1F). Double immunostaining showed that TLR8 was totally co-localized with the neuronal marker Tuj1 (Fig. 1G), but not with the macrophage marker CD68 (Fig. 1H), suggesting the neuronal expression of TLR8. The cell-size distribution showed that TLR8 was expressed mainly in small-diameter neurons (< 300 µm^2^, 70.8%), moderately in medium-diameter neurons (300 µm^2^–600 µm^2^, 27.8%), and rarely in large-diameter neurons (> 600 µm^2^, 1.4%, Fig. 1I). Further double staining showed that TLR8 was highly co-localized with the marker of neuronal injury ATF3 (Fig. 1J). Moreover, TLR8 was co-localized with the non-peptidergic marker IB4, and with the peptidergic marker CGRP, but rarely with the large-diameter neuronal marker NF200 (Fig. 1K–M). These data suggest that TLR8 is mainly expressed in small-diameter neurons in the TG and increases after pIONL.

### Deletion or Knockdown of *Tlr8* in the TG Alleviates pIONL-Induced Mechanical Allodynia

**Fig. 2:**
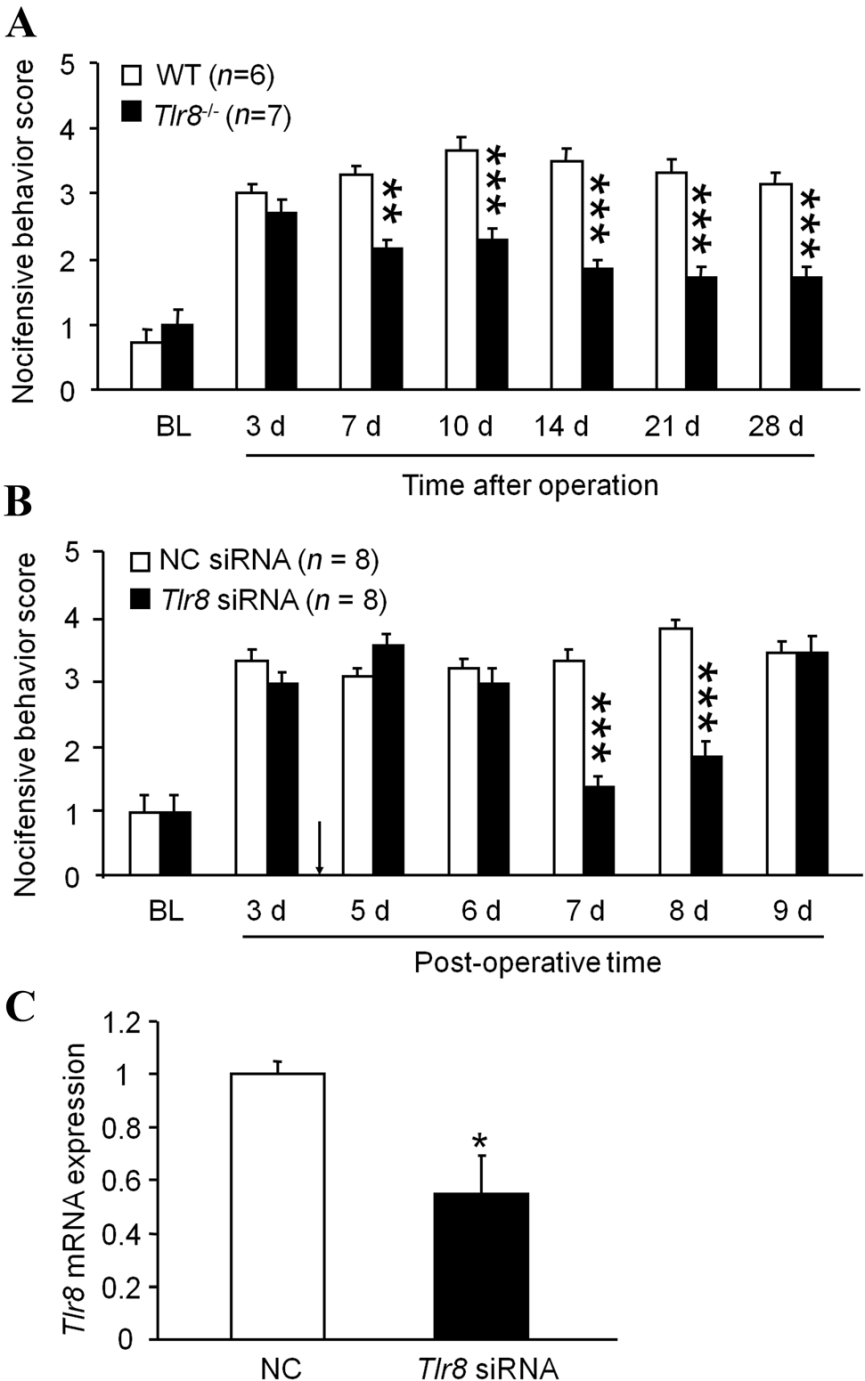
Global deletion of *Tlr8* or knockdown of *Tlr8* in the TG alleviates the mechanical allodynia induced by pIONL. **A** Nocifensive behavior scores stimulated by a 0.02 g von Frey filament in WT and *Tlr8*^−/−^ mice (**P < 0.01, ***P < 0.001 *vs* WT, two-way RM ANOVA followed by the Bonferroni test). **B** Nocifensive behavior scores stimulated by a 0.02 g von Frey filament after intra-infraorbital nerve injection of *Tlr8* siRNA mixed with RVG 4 days after pIONL (***P < 0.001 *vs* NC siRNA, two-way RM ANOVA followed by the Bonferroni test). **C** RT-PCR of TG tissue showing that *Tlr8* siRNA down-regulates *Tlr8* mRNA expression (*P < 0.05 *vs* NC siRNA, Student’s *t*-test; *n = *5 mice/group).

To determine whether TLR8 is involved in TNP, we compared the mechanical allodynia after pIONL in WT and *Tlr8*^−/−^ mice. As shown in Fig. 2A, the facial nocifensive behavior score evoked by a 0.02 g von Frey filament was significantly decreased from 7 days after pIONL until the end of the observation period in *Tlr8*^−/−^ mice, suggesting the alleviation of TNP after *Tlr8* deletion. To further check if TLR8 in the TG is involved in TNP, we specifically knocked down TLR8 expression in the TG by intra-infraorbital nerve injection of *Tlr8* siRNA mixed with RVG-9R [20]. The injection of *Tlr8* siRNA at 4 days after pIONL caused a decrease in the nocifensive score. The effect appeared at 3 days after the injection and was maintained for > 48 h (Fig. 2B). RT-PCR showed that *Tlr8* siRNA injection decreased the *Tlr8* mRNA level by 46.2 ± 14.7% after 24 h (Fig. 2C). These data suggest that TLR8 in the TG is involved in the maintenance of TNP.

### Deletion of *Tlr8* Reduces the pIONL-Induced Activation of ERK and p38, and the Expression of Pro-inflammatory Cytokines in the TG

**Fig. 3:**
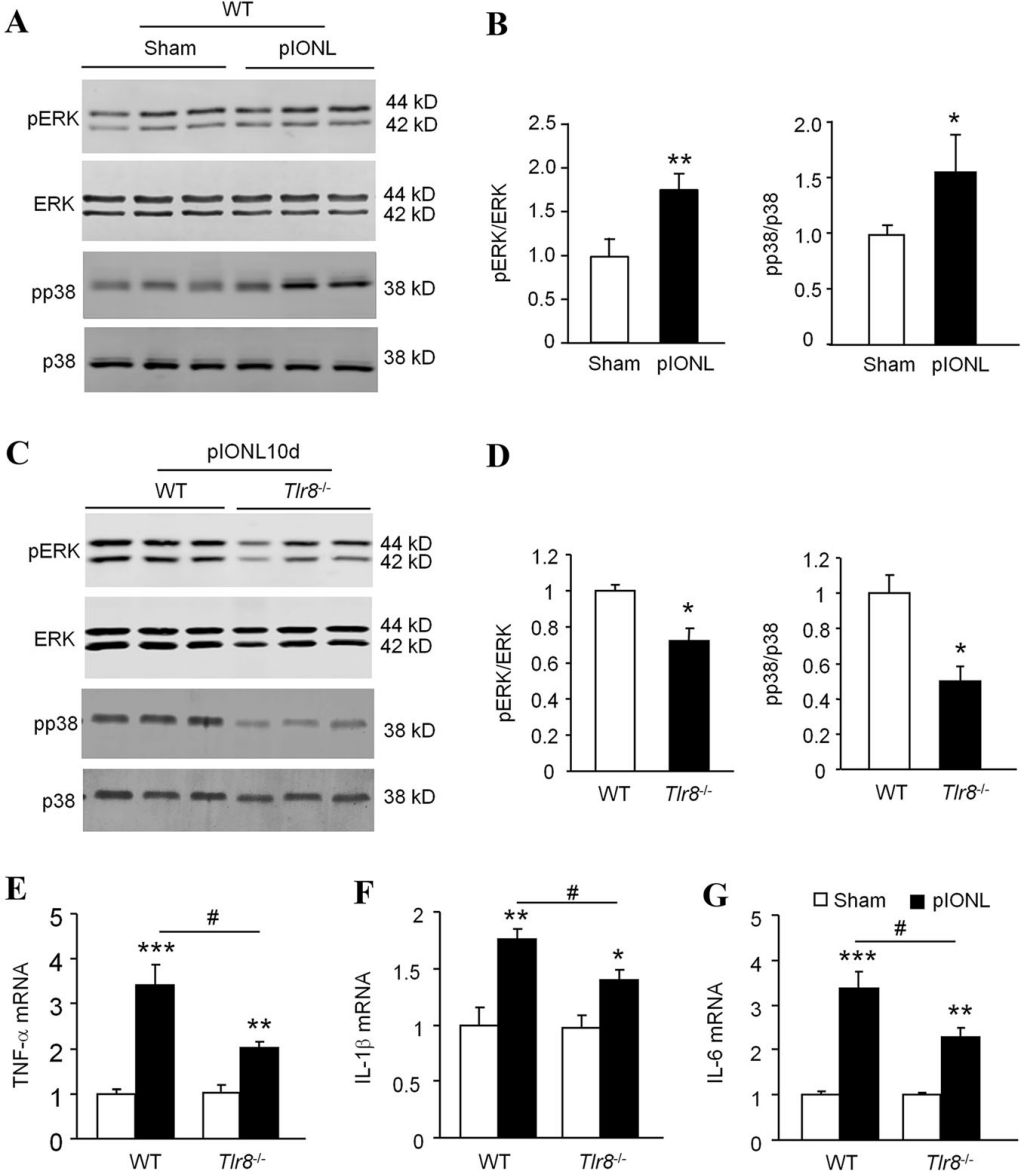
Expression of pERK, pp38, and pro-inflammatory cytokines induced by pIONL is attenuated in the TG of *Tlr8*^−/−^ mice. **A**, **B** Phosphorylation of ERK and p38 is increased at 10 days after pIONL in the TG of WT mice (*P < 0.05, **P < 0.01 *vs* sham, Student’s *t*-test; *n = *3 mice/group). **C**, **D** The phosphorylation of ERK and p38 induced by pIONL is reduced in the TG of *Tlr8*^−/−^ mice (*P < 0.05, *vs* WT, Student’s *t*-test; *n = *3 mice/group). **E–G** The expression of TNF-α (**E**), IL-1β (**F**), and IL-6 (**G**) is increased by pIONL in the TG of WT mice. The fold-increase of TNF-α (**E**), IL-1β (**F**), and IL-6 (**G**) induced by pIONL is lower in *Tlr8*^−/−^ mice than in WT mice (*P < 0.05, **P < 0.01, ***P < 0.001, pIONL *vs* sham; ^#^*P* < 0.05, *Tlr8*^−/−^ *vs* WT-pIONL, Student’s *t*-test; *n = *5–6 mice/group).

MAPKs have been well demonstrated in the pathological processes of neuropathic pain [21, 24, 25]. Our previous data showed that ERK and p38, but not JNK are activated in the TG after pIONL [24, 25]. Here we further confirmed the increased expression of pERK and pp38 in the TG 10 days after pIONL in WT mice (Fig. 3A, B). We then compared pERK and pp38 expression in WT and *Tlr8*^−/−^ mice and found that both pERK and pp38 level in the TG of *Tlr8*^−/−^ mice were lower than those in WT mice after pIONL (Fig. 3C, D). This suggests that TLR8 is involved in the pIONL-induced activation of ERK and p38 in the TG.

The pro-inflammatory cytokines TNF-α, IL-1β, and IL-6 have been demonstrated to play an important role in the development and maintenance of neuropathic pain [3, 20, 31]. Our real-time PCR data showed that pIONL increased the expression of TNF-α, IL-1β, and IL-6 in the injured TG of WT mice (Fig. 3E–G). In *Tlr8*^−/−^ mice, the average fold-increase of TNF-α, IL-1β, and IL-6 induced by pIONL was significantly lower than that in WT mice (Fig. 3E–G). These data suggest that pIONL-induced pro-inflammatory cytokine expression is partly dependent on TLR8 in the TG.

### Intra-TG Injection of TLR8 Agonist VTX-2337 Induces Pain Hypersensitivity

**Fig. 4:**
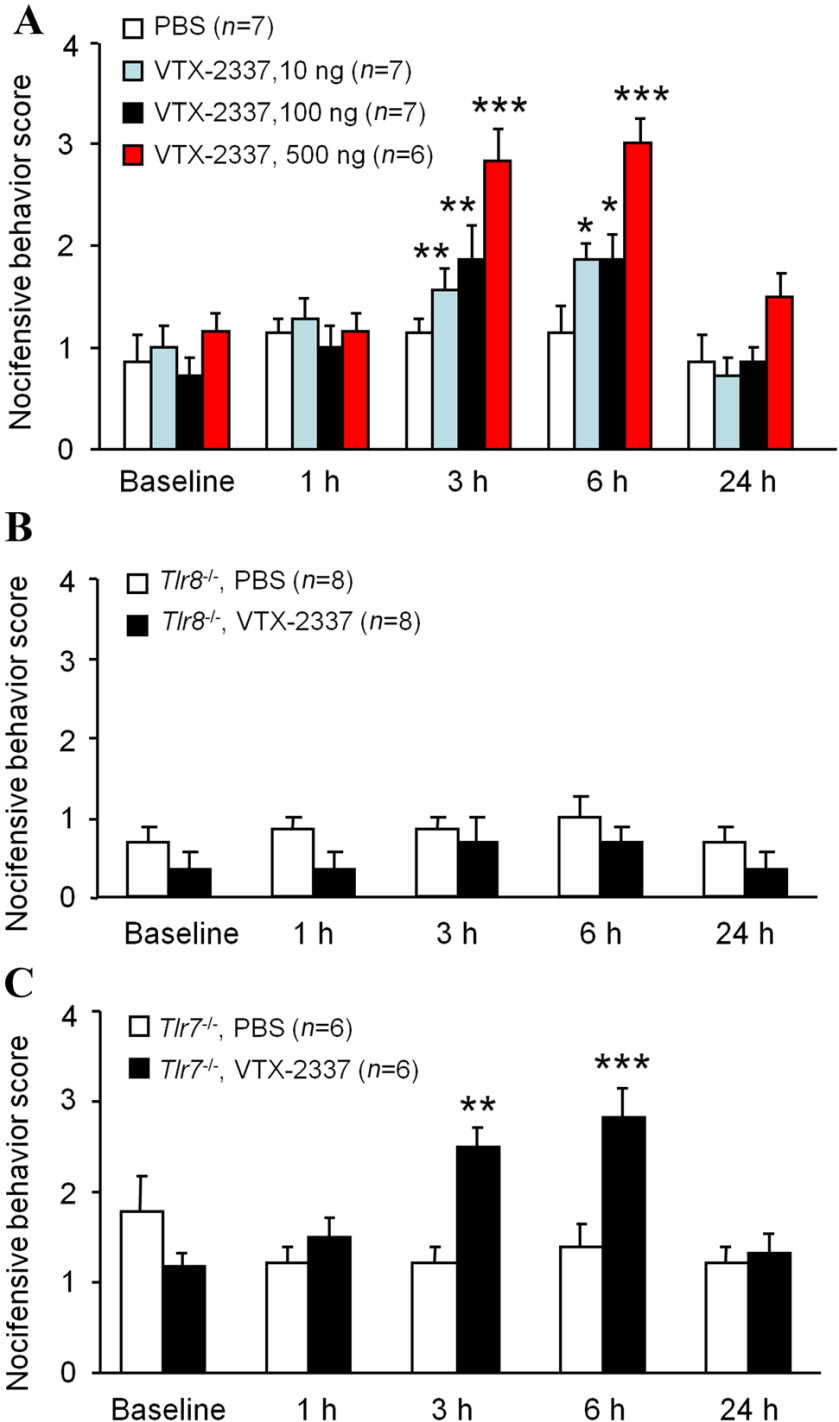
The TLR8 agonist VTX-2337 induces pain hypersensitivity in WT and *Tlr7*^−/−^ mice, but not in *Tlr8*^−/−^ mice. **A** Nocifensive behavior scores stimulated by a 0.02 g von Frey filament after VTX-2337 (10, 100, and 500 ng) or PBS injection into the TG in WT mice (*P < 0.05, **P < 0.01, ***P < 0.001 *vs* PBS, two-way RM ANOVA followed by Bonferroni test). **B** Nocifensive behavior scores stimulated by a 0.02 g von Frey filament after VTX-2337 (100 ng) and PBS injection in *Tlr8*^−/−^ mice. **C** Nocifensive behavior scores stimulated by a 0.02 g von Frey filament after VTX-2337 (100 ng) and PBS injection in *Tlr7*^−/−^ mice (**P < 0.01, ***P < 0.001 *vs* PBS, two-way RM ANOVA followed by Bonferroni test).

*Tlr8* and *Tlr7* are highly homologous, and both recognize viral single-stranded RNA. Several agonists including CL075, 3M-003, and R848 activate both TLR8 and TLR7 [32–34]. VTX-2337 is a selective and potent agonist of TLR8 [20]. We injected VTX-2337 into the TG to determine its effect on the pain hypersensitivity of the face. The behavioral data showed that intra-TG injection of VTX-2337 dose-dependently (50, 100, and 500 ng) increased the facial nocifensive behavior score (Fig. 4A). The pain hypersensitivity emerged 3 h after the injection and lasted for > 6 h. However, the intra-TG injection of VTX-2337 at 100 ng did not induce pain hypersensitivity in *Tlr8*^−/−^ mice (Fig. 4B). Despite this, the pain hypersensitivity was still evoked in *Tlr7*^−/−^ mice (Fig. 4C). These data indicate that TLR8 in the TG is sufficient to induce pain hypersensitivity on the face, and also demonstrate that VTX-2337-induced pain hypersensitivity is dependent on TLR8, not TLR7.

### TLR8 Agonist VTX-2337 Increases the Ca^2+^ Concentration in TG Neurons

**Fig. 5:**
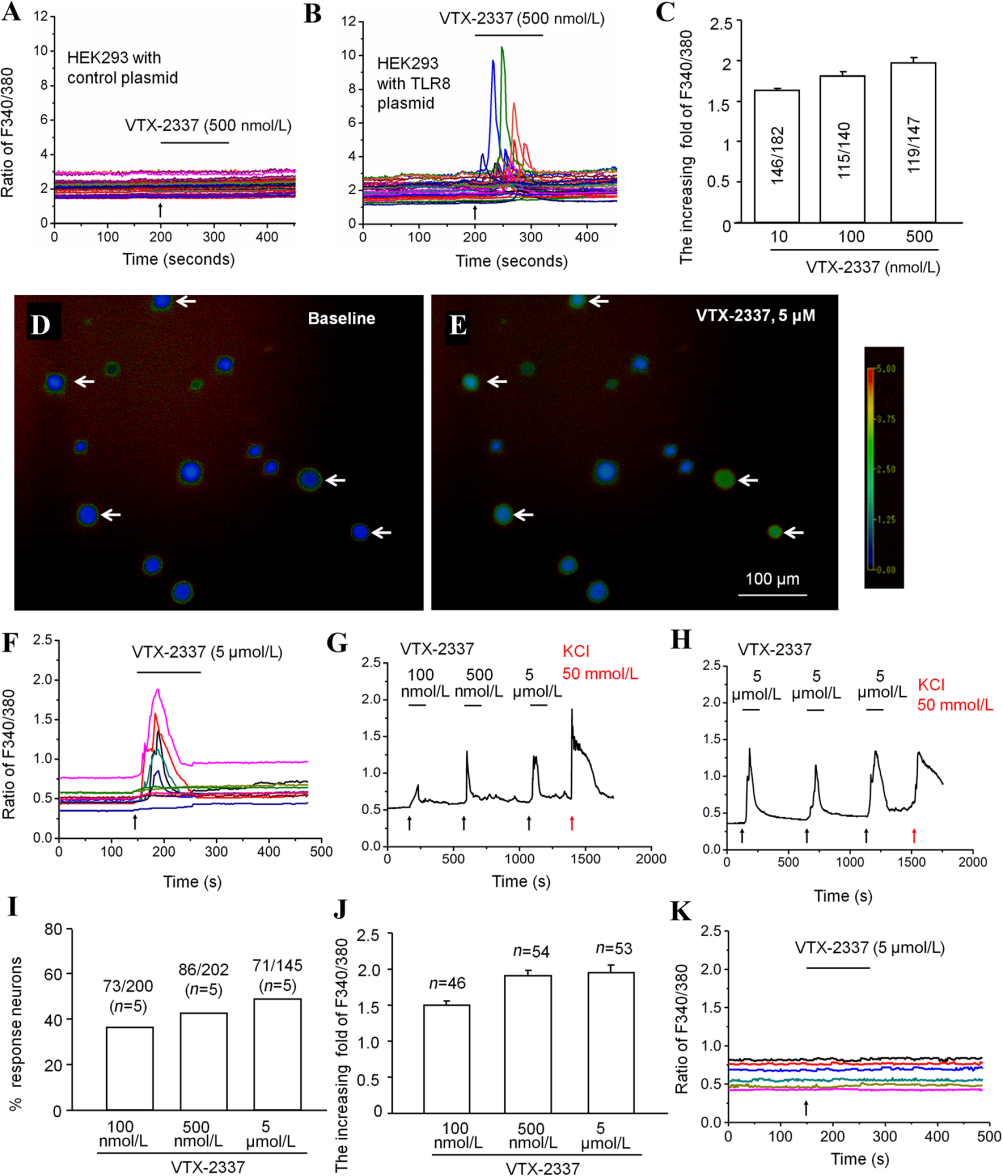
The TLR8 agonist VTX-2337 increases the Ca^2+^ concentration in HEK293 cells transfected with TLR8 plasmids and in TG neurons. **A**, **B** Effect of VTX-2337 on the F340/380 ratio in HEK293 cells transfected with control plasmids (**A**) or TLR8 plasmids (**B**). **C** Statistical data showing that VTX-2337 dose-dependently increases the F340/380 fold ratio in HEK293 cells transfected with TLR8 plasmids. **D**, **E** Representative Ca^2+^ images showing the intracellular Ca^2+^ activity in TG neurons at baseline (**D**) and with application of VTX-2337 (**E**) (arrows, neurons responding to VTX-2337). **F** Representative curves for the F340/380 ratio in TG neurons treated with VTX-2337 (5 μmol/L) in WT mice. **G** Representative trace of the F340/380 ratio in a TG neuron treated with VTX-2337 (100 nmol/L, 500 nmol/L, and 5 μmol/L) and KCl (50 mmol/L). **H** Representative trace of the F340/380 ratio in a TG neuron after three treatments with VTX-2337. KCl (50 mmol/L) is used as a positive control. **I** Percentages of neurons responsive to different concentrations of VTX-2337. **J** Fold increase of the F340/380 ratio after treatment with different concentrations of VTX-2337. **K** Representative curves of the F340/380 ratio in TG neurons in *Tlr8*^−/−^ mice treated with VTX-2337 (5 μmol/L).

As an important intracellular second messenger, intracellular Ca^2+^ ([Ca^2+^]_i_) is involved in many Ca^2+^-dependent intracellular signaling pathways. We checked whether VTX-2337 affects the Ca^2+^ response in HEK293 cells transfected with control or TLR8 plasmids. The Ca^2+^ imaging data showed that VTX-2337 had no effect on HEK293 cells transfected with control plasmids (Fig. 5A). However, VTX-2337 dose-dependently increased the F340/380 ratio of HEK293 cells transfected with TLR8 plasmids (Fig. 5B). With an increasing concentration of VTX-2337, the [Ca^2+^]_i_ also increased (Fig. 5C).

We further examined the effect of VTX-2337 on the [Ca^2+^]_i_ in primary TG neurons. The data showed that VTX-2337 at 5 μmol/L increased the F340/380 ratio (Fig. 5D–F). VTX-2337 at 500 nmol/L induced a [Ca^2+^]_i_ increase similar to that at 5 μmol/L, but 100 nmol/L VTX-2337 induced lower response (Fig. 5G). To test whether TG neurons desensitize to the repeated application of VTX-2337, we applied it three times at 500-s intervals, and the F340/380 ratio did not differ among the three applications (Fig. 5H). Statistical data showed that the percentage of responding neurons increased with increasing VTX-2337 concentration (Fig. 5I), and the fold-change of the F340/380 ratio was higher after treatment with either 500 nmol/L or 5 μmol/L than with 100 nmol/L VTX-2337 (Fig. 5J). Furthermore, we checked the Ca^2+^ response of TG neurons to VTX-2337 in *Tlr8*^−/−^ mice, and found no fold-increase in the F340/380 ratio after incubation with VTX-2337 (5 μmol/L) in *Tlr8*^−/−^ mice (Fig. 5K).

### TLR8 Agonist VTX-2337 Induces ERK and p38 Activation and Pro-inflammatory Cytokine Expression

**Fig. 6:**
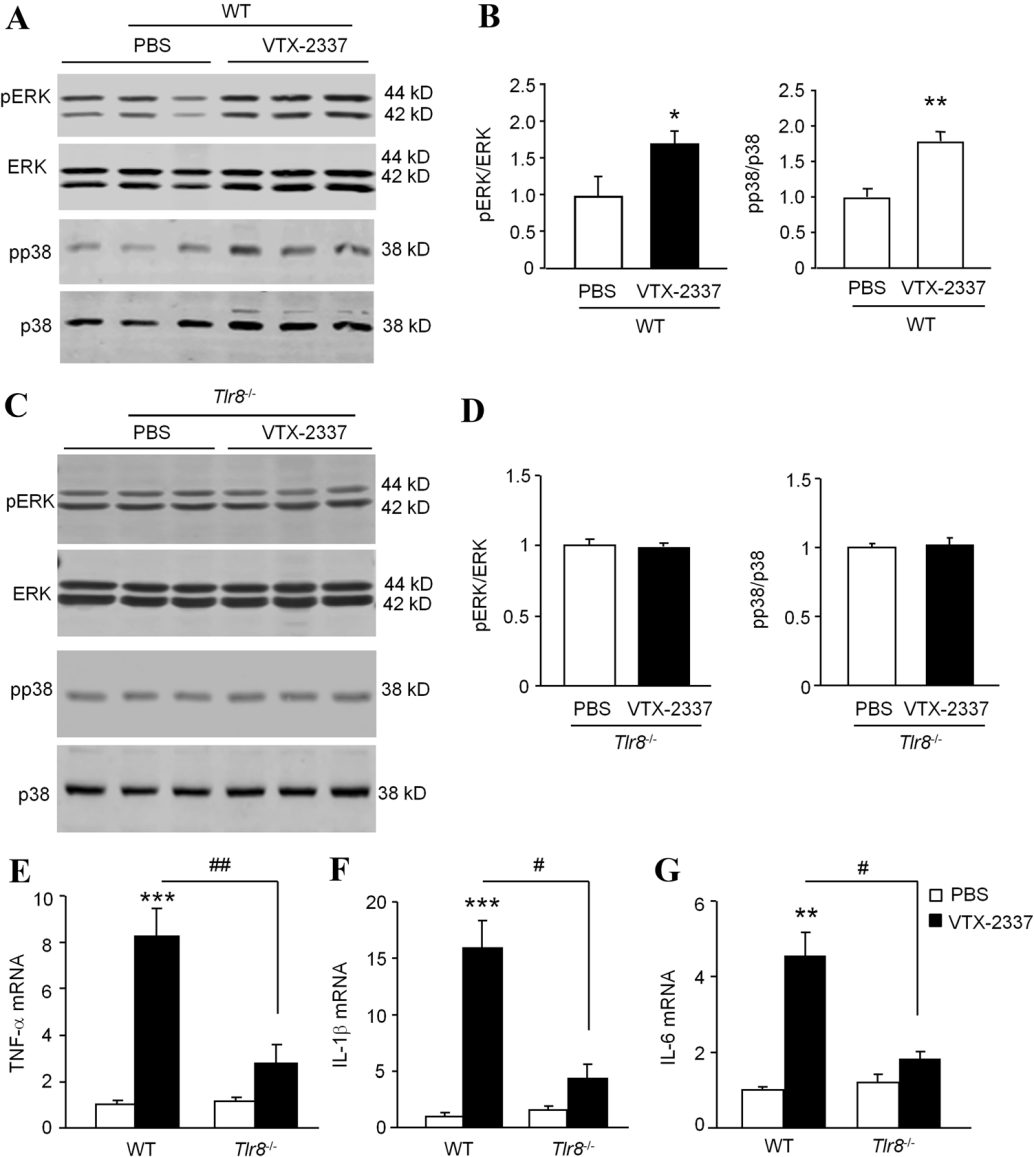
The TLR8 agonist VTX-2337-induced upregulation of pERK, pp38, and pro-inflammatory cytokines is reduced in *Tlr8*^−/−^ mice. **A**, **B** After intra-TG injection of VTX-2337, the phosphorylation of ERK and p38 is increased in the TG of WT mice (*P < 0.05, **P < 0.01 *vs* PBS, Student’s *t*-test; *n = *3 mice/group). **C**, **D** The phosphorylation of ERK and p38 in the TG does not increase after intra-TG injection of VTX-2337 in *Tlr8*^−/−^ mice (*P* > 0.05 *vs* PBS, Student’s *t*-test; *n = *3 mice/group). **E–G** The expression of TNF-α (**E**), IL-1β (**F**), and IL-6 (**G**) is increased by intra-TG injection of VTX-2337. The fold-increase of TNF-α (**E**), IL-1β (**F**), and IL-6 (**G**) induced by VTX-2337 is lower in *Tlr8*^−/−^ mice than in WT mice (**P < 0.01, ***P < 0.001 *vs* WT-PBS; ^#^*P* < 0.05, ^##^*P* < 0.01 *vs* WT-VTX-2337 group, Student’s *t*-test; *n = *5 mice/group).

To determine whether activating TLR8 can drive the activation of ERK and p38 in the TG, we injected VTX-2337 into the TG and checked the expression of pERK and pp38. Western blot showed that VTX-2337 increased the expression of pERK and pp38 in the TG of WT mice (Fig. 6A, B), but not in *Tlr8*^−/−^ mice (Fig. 6C, D). These data indicate that the activation of ERK and p38 expression induced by VTX-2337 is dependent on TLR8 in the TG.

To check the role of TLR8 in the expression of pro-inflammatory cytokines, we examined TNF-α, IL-1β, and IL-6 expression in the TG after intra-TG injection of VTX-2337 in WT and *Tlr8*^−/−^ mice. The real-time PCR revealed that VTX-2337 dramatically increased their expression in WT mice (*P* < 0.01 or 0.001 *vs* PBS, Student’s *t*-test), but not in *Tlr8*^−/−^ mice (*P* > 0.05 *vs* PBS, Student’s *t*-test). The fold-increase of TNF-α, IL-1β, and IL-6 in *Tlr8*^−/−^ mice induced by VTX-2337 was lower than that in WT mice (Fig. 6E–G), indicating that the VTX-2337-induced upregulation of pro-inflammatory cytokines is dependent on TLR8.

## Discussion

In this study, we showed that pIONL induced a persistent increase of TLR8 expression in small TG neurons, and the pIONL-induced pain hypersensitivity was alleviated in *Tlr8*^−/−^ mice and in WT mice treated with *Tlr8* siRNA in the TG. In addition, the pIONL-induced neuroinflammation, displayed as MAPK activation and pro-inflammatory cytokine production in the TG, was reduced in *Tlr8*^−/−^ mice. Consistently, the intra-TG injection of the TLR8 agonist VTX-2337 led to facial pain hypersensitivity, the activation of ERK and p38, and an increase of pro-inflammatory cytokine expression. These results suggest that TLR8 in TG neurons plays a critical role in the maintenance of TNP.

### TLR8 is Upregulated in IB4^+^ and CGRP^+^ TG Neurons After pIONL and Contributes to the Maintenance of TNP

TLRs, as one type of innate receptors, can recognize microbial pathogens and play an essential role in the initiation of innate immune responses [16]. In recent years, the role of TLRs in pain and itch has been widely investigated [17, 18, 35]. Several TLRs have been demonstrated to be expressed in glial cells and neurons in the DRG under chronic pain conditions [19, 36]. For example, TLR9 is expressed in DRG macrophages and contributes to chemotherapy-induced neuropathic pain [19]. TLR3 is expressed in small TRPV1-positive DRG neurons and regulates sensory neuronal excitability [37]. TLR7 is distributed in TRPA1-positive DRG neurons and is involved in both pain and itch [18, 38, 39]. Although it had been reported that TLR8 is non-functional in mice [40], increasing evidence has shown that TLR8 in immunocytes and neural cells has effects on inflammation and neuronal apoptosis [40, 41]. Our previous data showed that TLR8 is expressed in small IB4-positive DRG neurons and contributes to spinal nerve injury-induced neuropathic pain [20, 42].

In contrast to well-studied TLRs in the DRG underlying chronic pain, the expression of TLRs in the TG and their role in TNP are less studied. Here, we found that TLR8 had low expression in naïve mice but this dramatically increased in pIONL-operated mice. In addition, TLR8 was expressed in both IB4^+^ and CGRP^+^ small TG neurons after pIONL. As TLR8 was mainly expressed in IB4^+^ neurons in the DRG of naïve mice [20], we speculate that pIONL increases TLR8 in IB4^+^ neurons and induces TLR8 in CGRP^+^ neurons. Considering that IB4^+^ and CGRP^+^ neurons extend C- and Aβ-afferent fibers in the peripheral nervous system, and are widely recognized in the transmission of noxious signals to the spinal cord [20, 43], TLR8 may be involved in processing pain signals. Our behavioral data further showed that *Tlr8*^−/−^ mice suffered less mechanical allodynia than WT mice, and knockdown of TLR8 in the TG alleviated the TNP in the late phase (> 7 days), not in the earlier phase (< 3 days), suggesting the involvement of TLR8 in the maintenance of TNP. As TLR8 is expressed in primary afferents in the spinal cord [20], we do not exclude a possible role of TLR8 in the medulla oblongata in mediating TNP.

### TLR8 Contributes to the Maintenance of TNP *via* ERK and p38 Activation in the TG

In immunocytes, the canonical downstream signaling of TLR8 in endosomal compartments is associated with the production of cytokines mediated by the NF-κB pathway [17, 36]. However, the signaling pathway of NF-κB in peripheral sensory neurons cannot be activated by the TLR8 agonist VTX-2337 [20]. Instead, VTX-2337 induces ERK activation in the DRG after intrathecal injection, and spinal nerve ligation-induced ERK activation is also reduced in *Tlr8*^−/−^ mice, suggesting ERK as a downstream signal of TLR8 in the DRG [20]. It has been shown that ligation of the ION induces the activation of ERK and p38, but not JNK in the TG [24, 25]. ERK is also activated in TG neurons induced by migraine or lingual nerve crush [24, 44, 45]. We showed that the pIONL-induced ERK activation was reduced in *Tlr8*^−/−^ mice. Consistent with this, the intra-TG injection of VTX-2337 also induced pain hypersensitivity and pERK action in WT mice, but not in *Tlr8*^−/−^ mice. Similarly, the activation of p38 induced by pIONL was decreased in *Tlr8*^−/−^ mice, and p38 was also activated by VTX-2337 in a TLR8-dependent manner. Given that the inhibition of ERK activation by PD98059 (an MEK inhibitor) attenuates pIONL-induced mechanical allodynia and lingual nerve crush-induced pain hypersensitivity [24, 45], and the intra-TG administration of the p38 inhibitor SB203580 alleviates tongue pain and pIONL-induced TNP [25, 46], TLR8 may contribute to the maintenance of TNP *via* activation of ERK and p38 in the TG.

### TLR8 is Involved in Neuroinflammation

Neuroinflammation, which is characterized by the production of cytokines and chemokines in the nervous system, is one of the hallmarks in neuropathic pain [2, 3, 6, 7, 23, 47–49]. Several studies have shown increased levels of the cytokines TNF-α, IL-1β, and IL-6 in the DRG of animals with chronic pain induced by chemotherapeutic paclitaxel, chronic constriction of the sciatic nerve, experimental autoimmune encephalomyelitis, operation, or inflammation [22, 50–52]. The decreasing expression of TNF-α, IL-1β, and IL-6 in the DRG alleviates neuropathic and inflammatory pain [48, 51, 52]. Intra-TG injection of the TNF-α inhibitor etanercept, or the IL-1β inhibitor diacerein attenuate the pIONL-induced mechanical allodynia for up to 6 h [24]. In the present study, pIONL or intra-TG injection of VTX-2337 induced an increase of TNF-α, IL-1β, and IL-6 expression in the TG, and this was impeded in *Tlr8*^−/−^ mice, indicating that TLR8 is upstream of the pro-inflammatory cytokine production in the TG after pION.

Our previous studies showed that inhibition of ERK with PD98059 decreases the pIONL-induced upregulation of TNF-α and IL-1β in the TG [24]. The p38-MAPK inhibitor SB203580 also has a similar effect [25]. Therefore, activation of ERK and p38 in the TG may trigger the production of pro-inflammatory cytokines to induce TNP. *Vice versa*, pro-inflammatory cytokines activate the MAPK pathway to induce pain hypersensitivity in peripheral sensory neurons. In the DRG, IL-6 activates ERK signaling to induce pain hypersensitivity [53]. Likely, IL-1β also induces pain hypersensitivity through activating p38 in the DRG [54, 55]. Regional application of TNF-α at a nerve root causes pain hypersensitivity and increases pERK in small and medium DRG neurons [56]. Therefore, the activation of MAPKs induces the production of pro-inflammatory cytokines, which conversely boosts activation of the MAPK pathway in the peripheral sensory ganglia, such as the TG and DRG. This whole process creates a vicious cycle for neuropathic pain.

### TLR8 is Involved in the Increase of Intracellular Ca^2+^ and may Enhance Neuronal Excitability

Intracellular Ca^2+^ affects a wide variety of neuronal responses, including excitability. The excitability of primary sensory neurons increases after peripheral nerve injury [2, 23, 43]. Using Ca^2+^ imaging, we found that the TLR8 agonist VTX-2337 rapidly increased the [Ca^2+^]_i_ in HEK293 cells transfected with TLR8 plasmids and primary cultured TG neurons. The increased [Ca^2+^]_i_ may come from extracellular Ca^2+^ influx or intracellular Ca^2+^ release from organelles, such as the ER and lysosomes [10, 39, 57–60]. TLR8 is localized in the ER, endosomes, and lysosomes in human monocytes and macrophages [61, 62]. We recently demonstrated that TLR8 is also distributed in the same organelles in DRG neurons. Thus, we speculate that the increased Ca^2+^ after VTX-2337 treatment is released from intracellular stores. But how TLR8 mediates Ca^2+^ release needs further investigation.

It has been demonstrated that an increase of [Ca^2+^]_i_ can trigger a myriad of intracellular signals which include the phosphorylation of MAPKs (such as ERK and p38), inflammatory responses, and the release of neurotransmitters [57, 63, 64]. In addition, the activated ERK increases the expression of the Na^+^ channel Na_V_1.8, and regulates the TRPV1 activity in peripheral sensory neurons [65, 66]. Similarly, activated p38 also directly regulates the phosphorylation of Na_V_1.8 channels and increases the level of TRPV1 in DRG neurons [65, 67]. Furthermore, pro-inflammatory cytokines upregulate the expression of Na_V_1.7 and Na_V_1.6, further contributing to peripheral sensitization and neuropathic pain [68, 69]. Therefore, the activation of TLR8 may increase the excitability of TG neurons *via* regulating the expression and function of Na^+^ channels.

## Conclusion

In the present study, we demonstrated that pIONL increased TLR8 expression in small TG neurons. TLR8 may facilitate peripheral sensitization and trigeminal neuropathic pain through increasing intracellular Ca^2+^, activating MAPKs, increasing pro-inflammatory cytokine expression, and enhancing neuronal excitability. Thus, targeting TLR8 and its downstream signals in the TG may be a novel strategy for the treatment of TNP.
